# Practitioners of open dialogue report their personal transformations as a result of conducting network meetings

**DOI:** 10.3389/fpsyg.2022.1083996

**Published:** 2023-02-03

**Authors:** Alita Kathryn Taylor, Fletcher Brandon Taylor, Deborah Ann Anderson

**Affiliations:** ^1^Open Dialogue Pacific, Lynnwood, WA, United States; ^2^Department of Psychiatry and Behavioral Sciences, University of Washington, Seattle, WA, United States

**Keywords:** collaborative-dialogic approaches to mental healthcare, practitioner personal transformation, Open Dialogue network meeting, dialogical supervision, mutual transforming

## Abstract

As health care providers practicing Open Dialogue, we cleave to the notion that the support we provide to users and their communities will lead to the kind of enduring personal transformation that *they* would consider an improvement. But what effects do Open Dialogue network meetings have toward instilling enduring personal transformations within the practitioners themselves? This subject is rarely addressed, particularly in academic settings. In this autoethnographic account, an experiencer/occupational therapist, a marriage & family therapist, and a psychiatrist each describe enduring transformations that they attribute to working together as Open Dialogue network meeting facilitators at one stand-alone clinic over 2 years. Our report illustrates the potential of Open Dialogue network meetings, particularly the depth and breadth of transformation that can occur in *all* who attend them.

## Introduction

Like other post-modern approaches, Open Dialogue predicates second order cybernetics: In the act of observing and engaging with others during therapy, the helpers are inevitably changed by the process. There has been much care and attention given to exploring the allowing for, and the assimilation of utterances by clients during therapy (Anderson and Goolishian, 1992). The internal dialogue occurring *within* practitioners has also received attention often in the form of a microanalysis of therapy sessions *post hoc*, largely centered around the internal dialogue as recounted after a particular session with the help of taped recordings (Rober, 2005; Seikkula et al., 2012).

In our report, we explore the enduring transformations occurring within ourselves as practitioners attributing these transformations to our work with clients. In some ways this is new territory (Kahn and Fromm, 2001; Råbu et al., 2011; McNamee, 2015; Hirschhorn, 2016).

Such transformations deserve greater scrutiny since it is difficult to accompany clients in their journey any farther than we as practitioners have progressed in ours. Further, Carl Jung writes: “…the meeting of two personalities is like the contact of two chemical substances; if there is any reaction, both are transformed” (Jung, 1933).

In this autoethnographic account, an experiencer/occupational therapist, a marriage and family therapist, and a psychiatrist each describe enduring transformations that they attribute to working together as Open Dialogue network meeting facilitators at one stand-alone clinic over a two-year time period. Our accounts are as follows.

### Deb’s words

My primary response to being trained in Open Dialogue and participating in network meetings has been one of profound relief. Relief that I am not the only one who thinks this way, who wants to do work with others in this way, and who gets satisfaction out of using an open and supportive approach (Morasse, 2015; Sandmaier, 2019).

I am trained as an occupational therapist, having worked primarily in psychiatric settings, I also learned in my late 50’s how much of my experiences have in common with experiences labeled “psychotic,” and I found the psychiatric survivor movement. After that, I began training in Open Dialogue and fell in love.

Prior to learning how to work dialogically, professionally I had frequently struggled with the admonition to keep strict professional boundaries between my personal experiences and my professional role. While I could see that some sharing on the part of providers could lead to the focus coming off the person with the concern, I also knew that sharing oneself with others was a necessary requirement for developing relationships. I couldn’t understand why a professional relationship would be any different. As a young therapist with only a small amount of direct supervision, I realized that when I kept strict “professional boundaries,” this interfered with my ability to develop rapport with my clients.

Early in my career, I was shocked when one client told me they could share with me *because* I had crossed boundaries and shared my struggles. I then realized that careful sharing of my real self was actually more effective in helping to support behavioral changes than “treatment as usual.” The client was better able from my examples to understand the concepts being addressed and this then allowed them to act on those concepts to make changes to their own behavior. I began to notice more “aha!” moments in my clients when I shared, as opposed to when I did not. Indeed, the more frequently I judiciously shared my authentic experience, the stronger the bonds with my clients became and the more effective my interventions were.

This is consistent with polyphony, where all “voices” are welcome, whether they be internal or external, from client or professional, spoken or visual or behavioral. In network meetings, I experienced a sense of “opening” and interest when I spoke of my own experiences. One client in a network meeting spoke of his auditory hallucinations and then of feeling lonely. I asked if he wanted to meet people who also heard voices, and he agreed. His face lit up when I shared that I heard voices and offered to share my experiences with him.

I am incredibly moved by the interactive dance of words that takes place in network meetings. In training, I was taught that the professionals can ask to share their reflections with the clients. One family consistently requested that the professionals to share a reflection on what had just been said. It is not the “usual” or “expected” response, but in Open Dialogue, it’s all part of the conversation–there is no “one right way.”

After training and participating in network meetings over the past few years, I’ve noticed that my approach to conversations in therapy sessions, network meetings, and “real life” have changed. I am more likely to truly listen to what is being said without rehearsing my response while another is speaking. I seek to acknowledge with the person that I have understood them before going on. I am much more likely to ask a question than to respond with an answer. I more frequently bring curiosity to the conversation: What do you think about that? I wonder why (something happened)?

Personally, I feel more comfortable in social situations. I am much less likely to feel I need to say the “right” thing and I am more open to being in the moment and enjoying the conversation.

Professionally, I am more comfortable in sessions now that I can let go of having to be the “expert.” I enjoy the atmosphere of shared experiences rather than the traditional inequality of the professional’s (assumed) expertise as opposed to the (assumed) incompetence of non-professionals. This approach demonstrates respect for all in the room; it feels more intuitive and genuine, and it is almost uniformly acknowledged by the participants as being of value to them.

I feel honored to be in network meetings to witness and support participants in being their own genuine selves. By witnessing and accepting others in the context of network meetings, I have found I’m more likely to provide myself the same care and compassion that I give to clients.

### Fletch’s words

Much of our medical training, including psychiatry, centers around pattern recognition and applying treatment algorithms. We identify one or more diagnoses best fitting a constellation of symptoms followed by the adherence to treatment algorithms most likely to manage maladaptive behaviors in favor of adaptive ones. Arguably, a change toward adaptive behaviors is an orthodox standard of successful treatment (Coulacoglou and Saklofske, 2017). During our training as physicians the notion that positive personal transformation and attendant adaptive behaviors occurring in the clinician during the treatment of others is rarely discussed. The main interface between practitioner and patient within the Open Dialogue approach is the network meeting. In this context, I offer the following vignette of such personal transformation.

Over nine months’ time, the three of us (DA, FT, and AT) facilitated a series of network meetings, about two a month, involving an extended family concerned for a member, 25 year-old “Tom”, who said he had been hospitalized several times for paranoid schizophrenia. Accompanying Tom were his mother, father, four siblings, and his wheelchair-bound maternal grandmother. I was struck by the affectionate banter across three generations as well as their animated discussions on how to best support Tom as he struggled with self-care while alone in his apartment. There were times when Tom would storm out of the room, but he usually circled back a few minutes later after a smoke, having sorted himself out. We each commented at different times about the tension between he and his mother, how their conversations seemed stilted and awkward. When addressing this, Tom said he could not talk about it. Finally, during one meeting 3 months after beginning, the subject came up again and he turned to his mother and spoke: “Mom, you tried to poison me when I was 6 years old! Remember?”

The mother was mortified and said “I would never do that. How could you possibly think that I would do such a thing?”

“And why did you try to do a mind wipe?” He added.

The family struggled to reconcile these two vastly different versions of their history together. Each of them talked about their experiences around that time. Over the next few months, they all continued attending meetings, yet around this one issue there was never any literal/verbal reconciliation.

Later, someone reflected aloud: “I wonder what it’s like for mother and son to keep meeting together, to talk to each other when one is sure that the other has poisoned him?”

During and after the network meeting experiences above, I began to think how I could nudge myself toward an acceptance of the multiple realities within my own family members while still adhering to my core principles and sanity. I have a family member who in the home of my youth, as an adolescent, he had sexually abused another younger minor family member. For years, the perpetrator accused me of the offense that he alone committed. No one believed him. I held him in contempt, barely speaking to him for years.

For years, I had ignored his repeated calls and held him at arm’s length. Following these network meetings, I began answering his calls and we began to have tentative conversations about life. We met for lunch one day and we wound up discussing fatherhood. I watched myself listening to his concerns about raising his adolescent child while trying to set aside the deafening roar of my anger at his betrayal. Since then, we have become closer.

Thanks to Tom’s family and others like them, I have found within me a greater capacity to tolerate the viewpoint of someone whose stated realities and motives are not completely known to me. It is possible to set boundaries, respectful ones, while keeping open lines of communication with the understanding that we may never in this lifetime agree on some of the most basic things. If this kind of relationship is possible with a family member, it is possible with anyone.

My relationships with everyone have shifted in the direction of my possessing a more open mindset when others speak of their realities. I can listen to somebody’s opposing point of view and still hold firm to my most basic core value system. Somehow, as a result, I believe I have also become a bit less prideful. These are among the changes I have noticed since participating in the facilitation of Open Dialogue network meetings.

### Alita’s words

A career, if you’re lucky, should be something one endeavors with somewhat of a significant level of interest and engagement—that one can practice one’s own true nature, and perhaps even more wonderful, one’s own values. Psychotherapy/counseling was something I fell into as I joined my high-school on-campus peer counseling team at age 16. Talking to peers when they were in crisis seemed important and needed, something I thought I would want available for myself. So then, a “Judeo-Christian” value I was brought up in—you might say—“to treat others as you would like to be treated”— should not I make myself available to others in this way? I did. It led me to choosing to study psychology, thinking that in this way I could become a part of others’ healing; maybe even on a “soul” level. “Psyche” does indeed translate to soul. In my training, mental health practitioners are encouraged (and need I say it’s necessary) to do one’s own work in order that one learn to be present, capable, aware, and “do no harm,” as much as possible. I cannot control systems at large that govern the policies of mental health guidelines or implementation—(not directly or alone anyway). That said, I *can* continue practicing my values within the scope of psychotherapy, and even more so, I have found that to be the case by participating in network meetings.

Learning and practicing Open Dialogue, if I can even say it’s such a “thing,” {*rather, it’s an attitude or an idea held lightly; of doing less and “being with” more [as in a “benign expertise”* (Minuchin, 1998)]} has been something which in my career has, in a way, helped me to be more in myself and of myself *with* others at the same time. Promoting dialogue requires quieting oneself, leaving room for pondering, embodying an invitational silent presence for others to question and struggle together, to decide together how to go (Shotter, 1993). Dialogical practice cannot happen without my participation. It can also not happen in many moments when I attempt to control the outcome. This is (though arguable), philosophically, ontologically, for me, the *most* important. Dialogical practice *not* being about controlling the outcome is a transformative understanding to remember over and over, and over again with each family, each group/couple/business/team/meeting—that my *own* attempts to control or to be in charge of what *should* happen *when*, are not so much in dialogue with others.

We are human and we are born in dialogue, with nature, with other living things, and I cannot be unmoved. Open Dialogue network meetings are the medicine, not the doctor giving something to ail something wrong—we are co-creating space for language and understanding to emerge in its own way, and to be a witness, and to bring my body, attention, and time to be with the flow of the sharing of ideas. This is a radical way of being and also the most basic (McNamee, 2015).

I feel tremendous relief when co-facilitating network meetings because I don’t bring agenda, goals, needs for anyone to get better or get over some symptom. I keep my training in the background while attempting to stay fresh with each moment. This is how I would want to be met in crisis, and so I do my best to offer and create ways that network meetings can become standard for mental health healing endeavors. Transformation itself, too, is a living process, where my own changing is never done. Before calling myself an Open Dialogue practitioner, I might have thought that somehow there was an end to healing, that somehow my helping profession was solving something or someone, getting them “better.” I cannot unknow this collaborative-dialogical practice now. I carry lightly the helper role, remaining invitational to contexts, dilemmas re-naming themselves, allowing situations to be incomplete, placing the expert to be there between a person and their network themselves (i.e., the expert is *the relationship between a person and their network*). I have begun to learn what humility is, realizing that much of my career in emergency psychiatry *did* do harm, now, in offering and teaching others the history and practices of Open Dialogue, there might be, maybe, some reconciliation, for myself, and others, at the same time.

Thinking back over the start of my career serving psychiatric populations, I recall many times where I participated in care within a hospital setting where procedures and decisions made privileged the ease of systems and policy instead of the motto seen on posters about the hallways “*PATIENTS FIRST.”* Suffice it to say, “CYA” very often won out instead of us (hospital staff) risking to do what was called for, albeit inconvenient. Sometimes these memories flash before me, and there exists in me some sort of guilt, maybe for how I might have been seen by the eyes of colleagues, or acting from a place of fearing a bad work review or fear of losing my job if I stood up for the inconvenient patient’s way. I walked a narrow line at times, dare I say I buried this moral injury, some kind of by-stander effect, being a part of a system where human rights were not always honored, I tried to serve patients and their families, while feeling I had my hands tied behind on my back. It took a toll. Enter “open dialogue.”

Now in my own small practice, I still feel that guilt at times, or maybe it is pain, or lament, grief, for the so-many-others across time who’ve been met with fragmentation, disengaged from dialogue. And so how is one to be in dialogue with that? When I participate in the utterances of others in the meetings which I am a part of, truth emerges announced; dialogue is when there is a stream of meaning flowing among and through and between us (Bohm, 1996). How do we stay in that stream? I stay in it because not being in dialogue now feels like death. I want life. I want to live. Even if it is difficult, even if there is confusion about what I do or how a meeting will be, or how some care for a patient including a complicated network goes, it is alive, and it is dialogue, and I am open. Even to the strange or peculiar. Perhaps in the postmodern era of helping professions we will find in madness the wisdom that people of earlier ages found (Foucault, 2009).

## Conclusion

In the context of Open Dialogue network meetings all three of us practitioners attributed our personal transformations, at least in part, to what happened during network meetings. We agreed that our internal changes were profound enough to change our mindset and our behaviors, though our transformations themselves varied. We share our accounts in hopes that other practitioners, in making room for other voices, will continue to allow themselves to also be changed by them. The three of us were able to share these awarenesses for several reasons. The training that Deb and Fletch attended was presented in such a way as to encourage and allow this kind of self-reflection: we were encouraged to bring our own processes to discussions during the training itself. Inter-Vision (*our regular sessions together outside of network meetings which was a peer-based supervision/consultation format run dialogically*) allowed for further discussion and reflection among ourselves in an accepting and supportive environment. We were willing and available to have transdisciplinary conversations versus defending our own individual professional turf.

In conclusion, our participation in Open Dialogue network meetings has had a significant positive impact on each of us, professionally and personally. Deb significantly increased her scope of confidence in using open and supportive approaches and also was able to improve her own inner dialogue and be more helpful and understanding to herself. Fletch has used skills learned in network meetings to inform his professional approach, and has changed one family relationship from one of animosity on his part to being able to tolerate the vast differences between them and still be true to his own values. Alita has been able to let go of a sense of control to better support clients and to better live in alignment with her own values and ethics.

Network meetings can have a profound effect on all participants including the practitioners. We understand that one limitation of our autoethnographic accounts is how to reckon their applicability to the lives of other practitioners. As open dialogue service throughout the world continues to develop, further research on clinicians’ experiences in network meetings could lead to positive outcomes research pertaining to staff retention and quality of life in service systems, patient improvement and treatment satisfaction, and potentially to the reduction of burnout in the field of mental health in general. Ongoing dialogical supervision meetings and trainings are continuously needed to share our insights and new understandings for how practitioners find themselves changed in the process of conducting network meetings (Marovic and Snyders, 2010) remembering that transformation is a process; there is no end to our changing (Kunitz, 2007).

## Data availability statement

The original contributions presented in this study are included in the article/supplementary material, further inquiries can be directed to the corresponding author.

## Author contributions

All authors listed have made a substantial, direct, and intellectual contribution to the work, and approved it for publication.
